# On‐Surface Debromination of 2,3‐Bis(dibromomethyl)‐ and 2,3‐Bis(bromomethyl)naphthalene: Dimerization or Polymerization?

**DOI:** 10.1002/anie.202204123

**Published:** 2022-06-08

**Authors:** Yanning Tang, Barbara Ejlli, Kaifeng Niu, Xuechao Li, Zhengming Hao, Chaojie Xu, Haiming Zhang, Frank Rominger, Jan Freudenberg, Uwe H. F. Bunz, Klaus Muellen, Lifeng Chi

**Affiliations:** ^1^ Institute of Functional Nano & Soft Materials (FUNSOM) Jiangsu Key Laboratory for Carbon-Based Functional Materials and Devices Joint International Research Laboratory of Carbon-Based Functional Materials and Devices Soochow University Ren'ai road No. 199 Suzhou Jiangsu 215123 China; ^2^ Max Planck Institute for Polymer Research Ackermannweg 10 55128 Mainz Germany; ^3^ Organisch Chemisches Institut Ruprecht-Karls-Universität Heidelberg Im Neuenheimer Feld 270 69120 Heidelberg Germany; ^4^ Macao Institute of Materials Science and Engineering (MIMSE) MUST-SUDA Joint Research Center for Advanced Functional Materials Macau University of Science and Technology Taipa 999078 Macao China

**Keywords:** Conjugated Polymers, Dehalogenative Homocoupling, On-Surface Synthesis, Scanning Probe Microscopy

## Abstract

We describe the on‐surface dehalogenative homocoupling of benzylic bromides, namely bis‐bromomethyl‐ and bis‐*gem*‐(dibromomethyl) naphthalene as a potential route to either hydrocarbon dimers or conjugated polymers on Au(111). While bis‐*gem*‐(dibromomethyl) naphthalene affords different dimers with naphthocyclobutadiene as the key intermediate, bis‐bromomethyl naphthalene furnishes a poly(*o*‐naphthylene vinylidene) as a non‐conjugated polymer which undergoes dehydrogenation toward its conjugated derivative poly(*o*‐naphthylene vinylene) upon mild annealing. A combination of scanning tunneling microscopy, non‐contact atomic force microscopy and density functional theory calculations provides deep insights into the prevailing mechanisms.

## Graphical abstract

On‐surface reactions of benzylic bromides on Au(111) towards dimerization or polymerization via selective dehalogenative C−C coupling have been investigated. The unambiguous formation of conjugated poly(*o*‐naphthylene vinylene) and electronic structure after thermal induced dehydrogenation have been revealed.

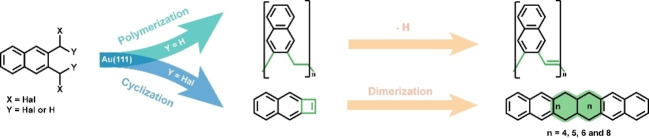

## Introduction

Thermally induced reactions performed after deposition of organic precursor molecules on metal surfaces under ultrahigh vacuum (UHV) conditions have emerged as a powerful addition to the toolbox of synthetic methods and to the study of unconventional modes of π‐conjugation.[^1^, ^2^, ^3^, ^4^] Biphenyl formation^[5]^ from on‐surface dehalogenative homocoupling of aryl halides proceeds via a homolytic cleavage of the carbon–halogen bond[^3^, ^6^] which differs from the electron‐transfer mechanism of the analogous Ullmann coupling in solution.^[7]^ On‐surface aryl–aryl coupling has recently attained special value since dihaloarenes offer an unprecedented access to polyarylenes.^[8]^ Further, the use of oligophenyl monomers with multiple twisted benzene units followed by thermally induced cyclodehydrogenation opens access to planarized graphenic structures such as graphene nanoribbons (GNRs).^[2]^

Although less common than aryl–aryl coupling, on‐surface reactions of *gem*‐dihaloolefins toward cumulenes and dihalomethyl arenes toward diaryl vinylenes have been studied as well.[^4^, ^9^, ^10^] Not surprisingly, 1,4‐bis(dibromomethyl)benzene can furnish poly(*p*‐phenylene vinylene) (PPV) chains which has found much attention in solution synthesis due to its electroluminesce.[^11^, ^12^, ^13^, ^14^, ^15^, ^16^, ^17^, ^18^, ^19^, ^20^, ^21^, ^22^, ^23^] The isomeric poly(*o*‐phenylene vinylene), however, has hitherto found less attention.[^24^, ^25^, ^26^] When halomethyl or dihalomethyl substituents are placed in the *ortho*‐positions of an arene, the analogous formation of polymers can compete with intramolecular C−C bond closure, possibly followed by dimerization. Despite the competition, the reactivity of halogenated *ortho*‐dimethylarenes in on‐surface reactions has so far escaped attention. By contrast, the brominated *ortho*‐dimethylarene derivatives have attracted much interest in solution reactions with either nucleophiles or metals.[^24^, ^26^, ^27^, ^28^, ^29^, ^30^, ^31^, ^32^, ^33^, ^34^]

Herein, we investigate the on‐surface reactions of di‐ and tetra‐brominated 2,3‐dimethylnaphthalenes (**DBN** and **TBN**) on Au(111) surfaces under ultra‐high vacuum conditions. Naphthalene is chosen instead of the benzene core to lower the volatility of the samples on the metal surfaces. The on‐surface debromination of **TBN** appears to afford different dimeric hydrocarbon products, while that of **DBN** gives poly(*o*‐naphthylene vinylidene) as a non‐conjugated polymer which upon further mild annealing undergoes dehydrogenation toward the conjugated poly(*o*‐naphthylene vinylene). By monitoring the reactions with scanning tunneling microscopy (STM), non‐contact atomic force microscopy (nc‐AFM), scanning tunneling spectroscopy (STS) and density function theory (DFT) calculations, the prevailing reaction mechanisms are disclosed. Most importantly, the emerging selectivity of intra‐ vs. intermolecular C−C bond formation is discussed by identifying the key intermediates whose interaction with the metal surface plays a critical role.

## Results and Discussion

**Scheme 1:**
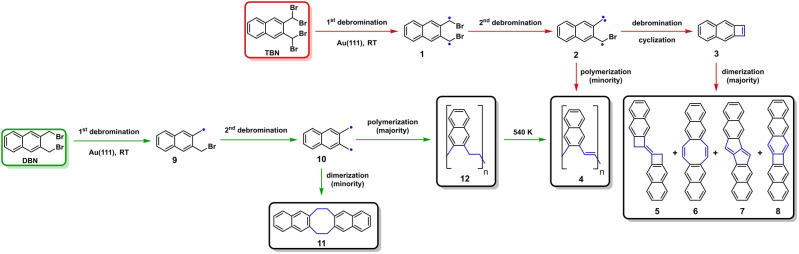
Schematic illustration of reactions on Au(111). 2,3‐Bis(dibromomethyl)naphthalene (**TBN**) (red); 2,3‐bis(bromomethyl)naphthalene (**DBN**) (green).

**Figure 1:**
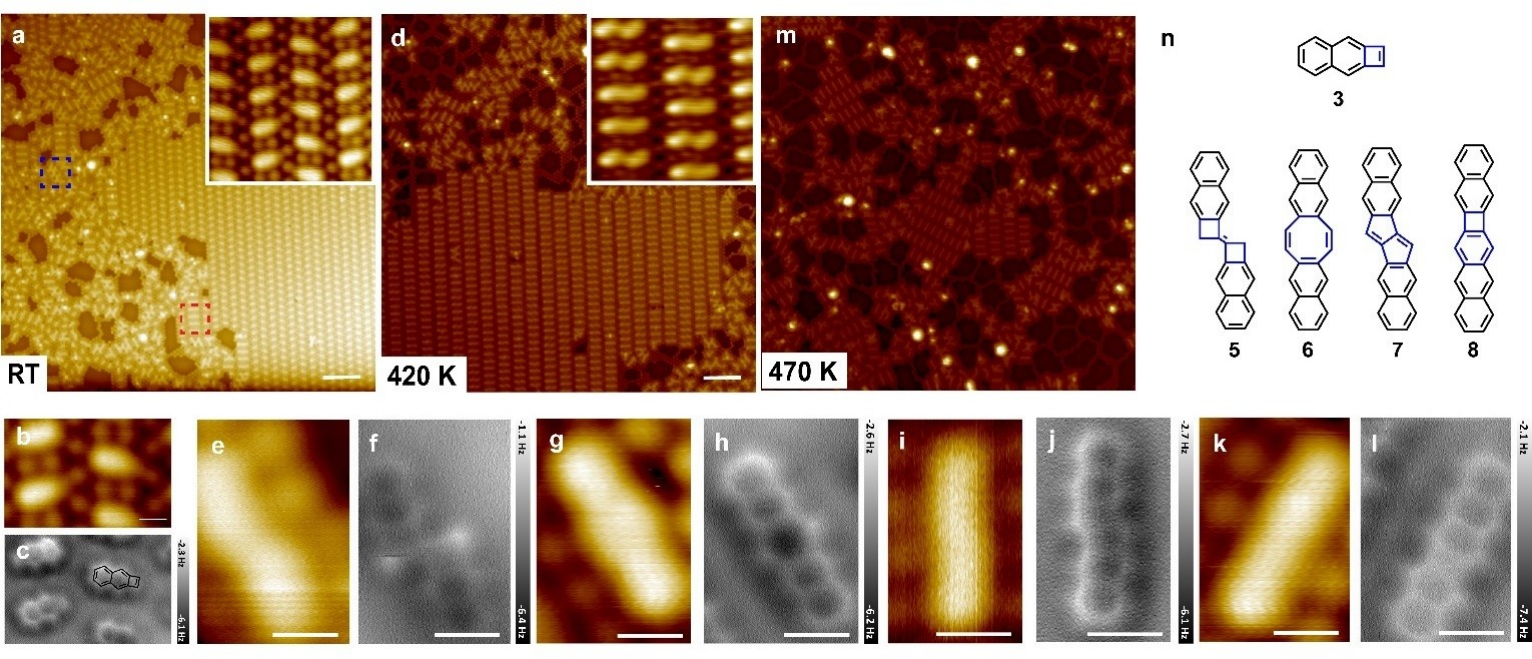
The products of **TBN** on Au(111) surface. a) STM image of **TBN** deposited on Au(111) at RT (*I*=5 pA, *V*=200 mV). Two dashed squares mark the area of minor structures, dimer (red square) and oligomeric species (blue square) respectively. Inset: A zoom‐in STM image of the monomer domain. b) A high‐resolution STM image of **3** surrounded by bromine atoms. c) nc‐AFM image of **3** with overlaid structural model. d) STM image of the sample after annealing at 420 K (*I*=5 pA, *V*=−0.20 V). A high‐resolution zoom‐in STM image is inset in d) (*I*=500 pA, *V*=−1 V). e), f) STM and AFM images of **5**. (*I*=3 pA, *V*=−0.20 V, Δ*Z*=−175 pm), g), h) STM and AFM images of **6**. (*I*=3 pA, *V*=−0.20 V, Δ*Z*=−150 pm), i), j) STM and AFM images of **7**. (*I*=3 pA, *V*=−0.20 V, Δ*Z*=−190 pm), k), l) STM and AFM images of **8**. (*I*=3 pA, *V*=−0.20 V, Δ*Z*=−185 pm), m) STM image of the sample after annealing at 470 K (*I*=5 pA, *V*=−0.20 V). n) Structural models of **3**, **5**, **6**, **7** and **8**. The white scale bars are 5 nm in (a), (d), (m), and 0.5 nm in the rest STM/nc‐AFM images.

2,3‐Bis(dibromomethyl)naphthalene (**TBN**) and 2,3‐bis(bromomethyl)naphthalene (**DBN**) were synthesized (Supporting Information, Figure S1–S6) as for on‐surface reactions by using a modified Wohl–Ziegler bromination of 2,3‐dimethylnaphthalene with *N*‐bromosuccinimide and azobis‐(isobutyronitrile).^[34]^ Because of their relatively high vapor pressure at room temperature, these precursor molecules were loaded in a commercial Knudson cell evaporator (Kentax, Germany) separately pumped under an ultra‐high vacuum (UHV) environment. The deposition on clean Au(111) surface was achieved in a preparation chamber with a base pressure better than 3×10^−10^ mbar. An overview over the complex reaction pathways observed after depositing both molecules on Au(111) is presented in Scheme 1 and now discussed in detail. When depositing **TBN** on Au(111) held at room temperature (RT), the molecules mainly assemble as “monomers” into extended domains (Figure 1a). Close‐up STM images (inset Figure 1a and Figure 1b) display unevenly shaped species surrounded by regular dots of dissociated Br atoms. Further, a high resolution nc‐AFM image (Figure 1c) allows to identify the planar product **3** as a naphthocyclobutadiene (complementary STS measurements of **3** are provided in the Supporting Information by Figure S7). A minor portion of short oligomerization and dimerization products (Figure 1a, marked in blue and red rectangles) can be detected at room temperature as well. Upon annealing to 420 K, dimeric structures prevail and increase in yield.

They self‐assemble into regularly packed domains (Figure 1d), accompanied by only a few oligomeric chain structures. The inset of Figure 1d discloses four different dimerization products. High resolution STM and nc‐AFM images (Figure 1e to 1l) uncover that these dimers can be categorized into two sets of constitutional isomers (**5**, **6**) (direct dimerization product of **3** shown in Figure 1e–h) and (**7**, **8**) [further dehydrogenation products of (**5**, **6**), shown in Figure 1i–l] in Scheme 1. The dimer structures thus represent the following sequences of rings: **5**: (6‐6‐4‐4‐6‐6), **6**: (6‐6‐8‐6‐6), **7**: (6‐6‐5‐5‐6‐6) and **8**: (6‐6‐6‐4‐6‐6). This remarkable complexity has seen no equivalence in the corresponding solution chemistry.

The relative amounts of these structures depend upon the annealing temperature: at 420 K, compounds **7** (54 %) and **5** (42 %) are by far the majority products with minor amounts **6** and **8**. By further annealing of the sample to 470 K, **5** is suppressed as well and **7** is the dominant product on the surface. A statistical analysis counting over 1000 dimers reveals that dimer **7** is the favored on, both, the pre‐heated surface and progressively‐heated sample (Supporting Information, Figure S8) which suggests that this configuration is thermodynamically favored on Au(111).

**Figure 2:**
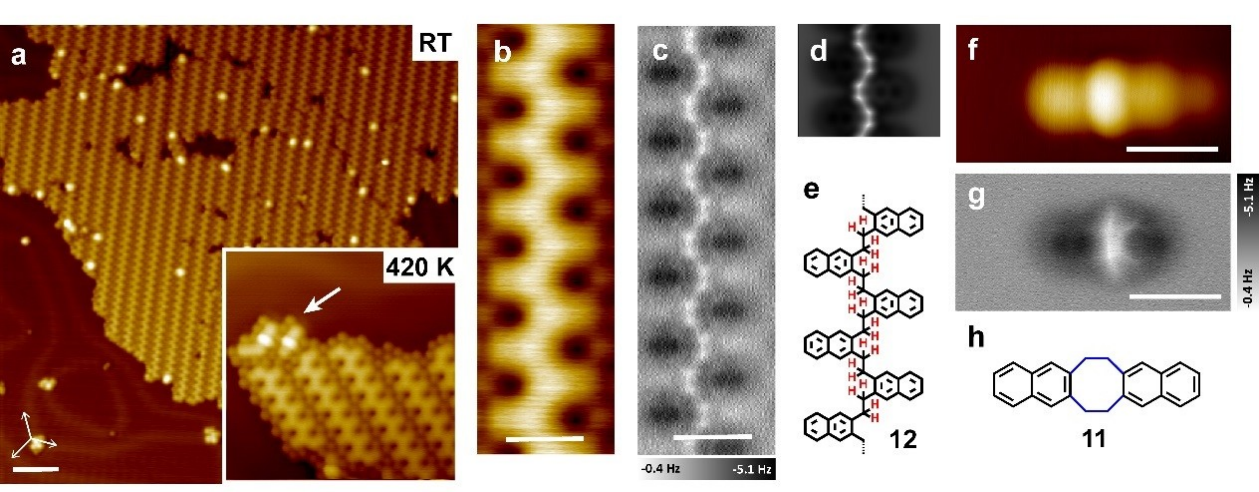
The products of **DBN** on the Au(111) surface. a) STM image of **DBN** deposited on Au(111) at RT (*I*=5 pA, *V*=200 mV). Inset: A magnified STM image highlighting dimers **11** after annealing to 420 K. b), c) High‐resolution STM and AFM image of poly(o‐naphthylene vinylidene) 12. (*I*=5 pA, *V*=200 mV, Δ*Z*=−150 pm), d) AFM image simulation of **9**, Δ*f* tip‐Z=−7.3 Å. e) Structural model of **12**. f), g) High‐resolution STM and AFM image of **11**, obtained after annealing 520 K. (*I*=5 pA, *V*=200 mV, Δ*Z*=−150 pm), h) Structural model of **11**. The white scale bars are 5 nm in (a), and 1 nm in the rest STM/nc‐AFM images.

The thermally promoted reaction of **DBN** on Au(111) takes a different course. With low coverage at RT, the fully debrominated intermediate state **10** is captured by STM (Supporting Information, Figure S9). Upon increasing the coverage, the majority of molecules spontaneously undergoes polymerization, forming well‐aligned parallel chains (Figure 2a) supposed to be poly(*o*‐naphthylene vinylidene) (**12**). When annealing the sample to 420 K, small features with bright protrusion in the center (highlighted with an arrow in the inset of Figure 2a) are occasionally observed at the periphery of the domains. To clarify the precise structure of **12**, high resolution nc‐AFM measurements were conducted, as presented in Figure 2c. The connection between naphthalene moieties is supported by nc‐AFM imaging as C(sp^3^)−C(sp^3^) bond formation (as the model in Figure 2e shown), whereby the dot‐like features in the frequency shift image serve as the fingerprint of alkanediyl units.^[35]^ Simulations of the AFM images are performed with Probe Particle Model (PPM) software (see Figure 2d) which agree well with nc‐AFM imaging. Figure 2g allows to also identify the minor dimer as non‐planar dinaphthotetrahydrocyclooctane (**11**). The magnified images (inset in Figure 2a, Figure 2f) uncover that **11** and **12** are surrounded by chemisorbed bromine (Br) atoms dissociated from **DBN**. Further depositing precursors onto the pre‐heated surface favours dimerization over polymerization (Supporting Information, Figure S10).

**Figure 3:**
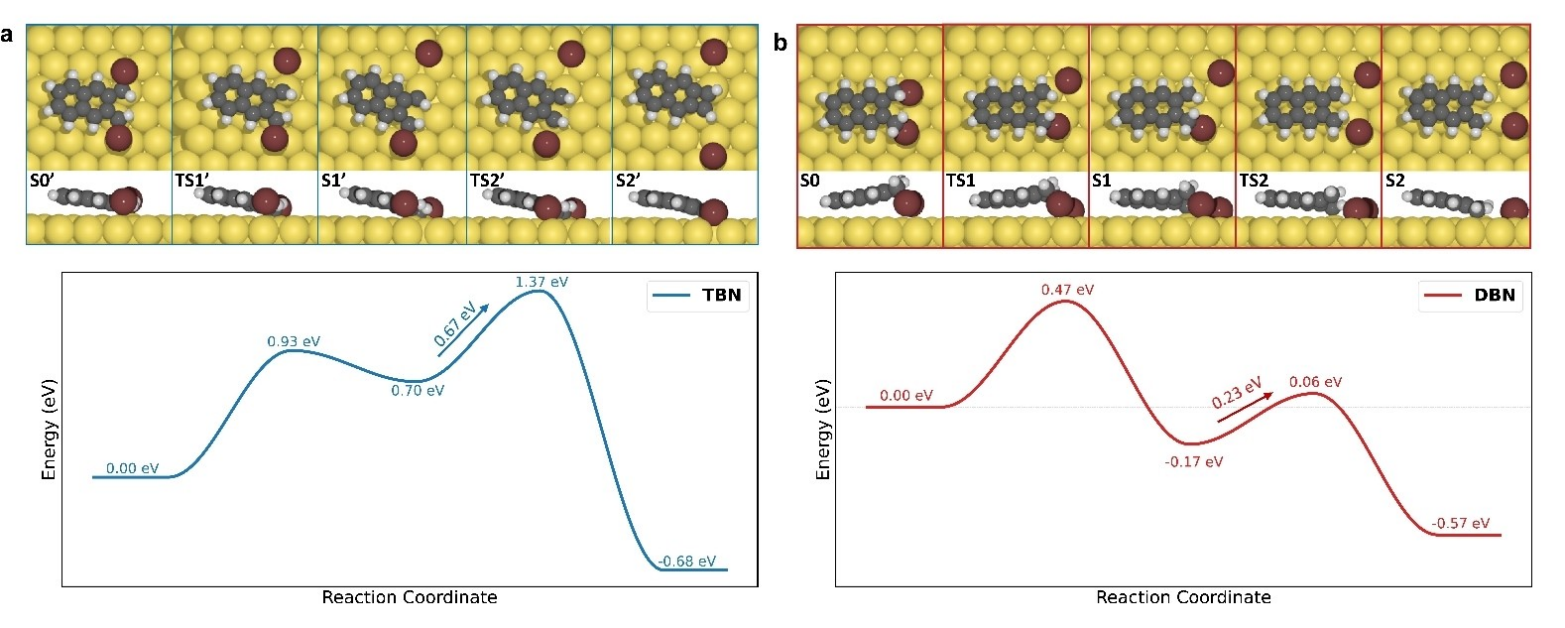
The debromination pathways and corresponding energy profiles of a) **TBN** (labelled in blue) and b) **DBN** (labelled in red). The Au, Br, C and H atoms are represented by yellow, brown, gray, and white circles, respectively.

To understand the different reactivity of **TBN** and **DBN** after debromination, the reaction mechanisms have been investigated by DFT calculations (detailed methods are described in the Supporting Information). First, dibromo‐substitution, instead of the monobromo‐substitution, at a methyl group enhances reactivity and excludes adsorption of intact molecules. Therefore, the simulation of **TBN** must commence with the adsorption of species **1** having already undergone loss of two Br atoms upon contact with the Au(111) surface at RT (**S0′** in Figure 3a). The further debromination is accompanied by a spontaneous cyclization step to generate the four‐membered ring of naphthocyclobutadiene (**3**), instead of surface‐stabilized C−Au bonds or *o*‐quinodimethane species. On the other hand, the reactivity of **TBN** clearly differs from that of 1,4‐bis(dibromomethyl)benzene for which the barrier for formation of poly(*p*‐phenylene vinylene) on the surface appears to be very low.^[9]^ The intermolecular coupling pathway is thus not favoured for **TBN** due to the rapid ring closure that cannot be overruled by other thermodynamically allowed processes.

In case of **DBN**, the dissociation of a C−Br bond in **DBN** starts from the physiosorbed state **S0** with an activation energy of 0.47 eV, leaving a chemisorbed Br atom on the hollow site of Au(111) and a surface‐bound intermediate **S1** (Figure 3b). After a similar cascade debromination, “surface‐stabilized radicals” are formed on Au(111) in which the C−Br bonds are replaced by C−Au bonds. Further DFT calculations demonstrate that the debrominated intermediate **S2** (**10**) resulting from **DBN** is so strongly adhering to the surface that intermolecular coupling becomes impossible at room temperature (Supporting Information Figure S11). This outcome, however, stands in contrast to the experiment, probably due to unrevealed kinetic effects during the surface‐stabilization of radicals.

As reported in on‐surface Wurtz reactions,^[36]^ the *anti*‐conformation (which suffers much less steric hindrance upon further polymerization) is energetically favoured over the *syn*‐conformation. The experimentally observed room temperature polymerization might be caused by the fast reaction kinetics with instantaneous charge redistribution as proposed by Polanyi and co‐workers.^[37]^ Indeed, one cannot rigorously exclude that intermolecular coupling between mono‐debrominated intermediates (**2** and **9**) occurs and, thus, contributes to the experimentally observed selectivity. Though consecutive debromination under adiabatic conditions appears reasonable, a clear discrimination of simultaneous or consecutive debromination is impossible without femtosecond time‐resolved measurements.^[38]^ Another possible explanation is that two Br atoms possess different chemical environments upon adsorption, as reported in the case of 9,11‐dibromonaphtha[1,2,3,4‐*ghi*]perylene adsorbed on Cu(111) and NaCl bilayers.^[39]^

Different from the initial expectation and different from the analogous benzene and naphthalene cases in solution,[^24^, ^26^, ^27^, ^28^, ^29^, ^30^, ^31^, ^32^, ^33^, ^34^] the tetra‐brominated precursor **TBN** does not tend to form the conjugated polymer **4** on surface. The present studies, however, reveal an alternative approach to poly(*o*‐naphthylene vinylene) by dehydrogenating the non‐conjugated polymer **12** obtained from **DBN**. In an independent study, we have uncovered the transformation of *n*‐alkanes to fully conjugated polyenes at elevated temperature on Cu(110) surfaces.^[40]^ By introducing an aromatic group (e.g. benzene or naphthalene) as the anchoring group, the dehydrogenation at the benzylic methylene groups proceed with a significantly reduced activation barrier.^[40]^

**Figure 4:**
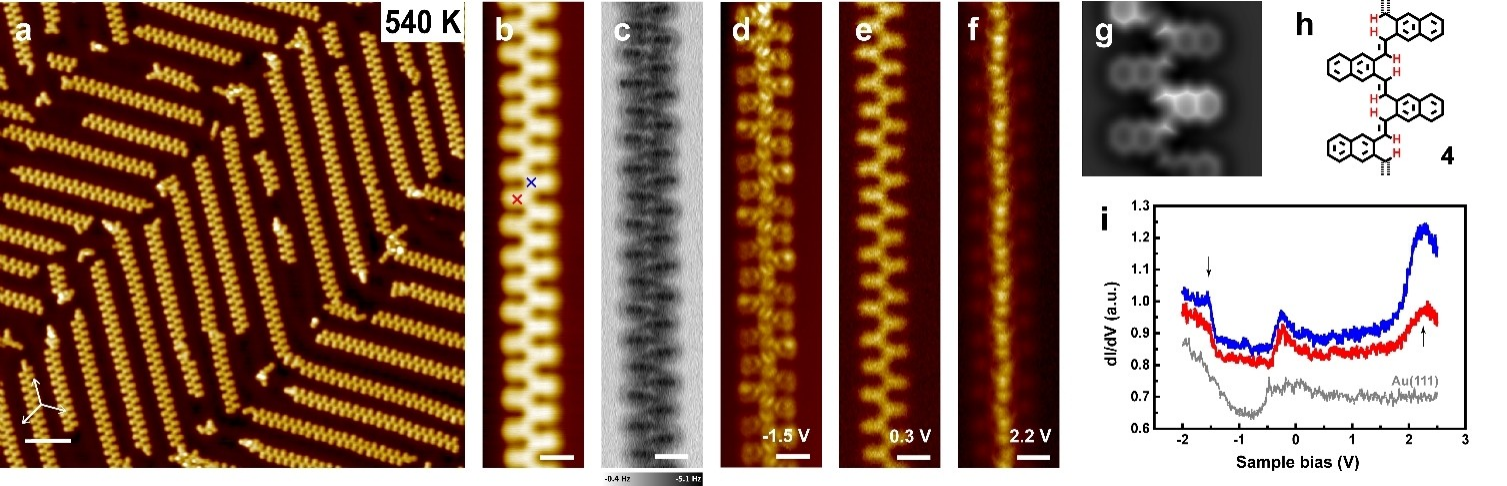
Reaction products of **DBN** on Au(111) surface under 540 K annealing. a) STM image of poly(*o*‐naphthylene vinylene) 4. b), c) High‐resolution STM and AFM image of **4**. d)–f) Constant height d*I*/d*V* mapping of **4** taken at sample bias voltage of d) −1.5, e) 0.3, and f) 2.2 V, respectively. g) AFM image simulation of **4**, Δ*f* tip‐*Z*=−7.3 Å. h) Structural model of **4**. i) d*I*/d*V* spectra of polymer **4** and bare Au(111), tip positions were marked in colored crosses in (b). The white scale bars are 5 nm in (a), and 1 nm in the rest images.

Here, further heating of **12** on Au(111) to 540 K causes desorption of Br atoms, leaving dispersed parallel chains (Figure 4a) which are then identified as poly(*o*‐naphthylene vinylene) (4). The fine structural details are revealed by high resolution STM and nc‐AFM displayed in Figure 4b and c which illustrate the replacement of vinylidene by vinylene fingerprints.^[9]^ The naphthalene cores are now lying at the same height as the vinylene connection and the structures become planar. AFM image simulations (Figure 4g) agree well with experimental observation. We thus successfully demonstrate that our concept concerning the conversion of alkanes to polyenes works on the less reactive surface such as Au(111) as well, reducing the stereospecifically transformation of the vinylidene polymer **12** into poly(*o*‐naphthylene vinylene) (**4**).

Poly(*p*‐arylene vinylenes) have been amply studied in terms of their extended conjugation and effective conjugation length which features have been essential for an understanding of their photophysical properties.[^11^, ^12^, ^14^, ^15^, ^16^, ^17^, ^18^, ^19^, ^20^, ^21^, ^22^, ^23^, ^41^] A comparison with the corresponding ortho‐analogues will have to consider, in particular, the effect of steric hindrance upon conjugation and chain conformation. For understanding the electronic structure of poly(*o*‐naphthylene vinylene) (**4**), STS measurements were conducted (see Figure 4i). The differential spectra obtained for **4** (blue and red lines) and bare Au(111) (grey line) are presented for comparison. Spectra taken with the tip position on the core (red) and vinylene connection (blue) of **4** show no differences in general. In both curves, two prominent resonance peaks are observed, centered at −1.5 V and 2.2 V which can be assigned to the Highest Occupied Molecular Orbital (HOMO) and the Lowest Unoccupied Molecular Orbital (LUMO) states, respectively (Figure 4i). A weak resonance centered at −0.3 V is recognized as a shifted surface state. Constant height d*I*/d*V* mappings at each resonance bias are collected and shown in Figure 4d–f. The spatial distribution of electronic states under negative bias proves the conjugated nature of polymer **4** in which the electrons fill in homogeneously, while under positive bias the electronic states are centered at the vinylene bridges. A direct comparison with on‐surface synthesized PPV in electronic properties is unfortunately not possible, since the STS studies failed to give characteristic features.^[9]^

## Conclusion

We have demonstrated the dehalogenative C−C coupling of 2,3‐bis(bromomethyl)‐ and 2,3‐bis(dibromomethyl)naphthalene on Au(111) surfaces which generates a variety of different structural motifs. The complex processes and the reaction mechanism have been studied by a combination of STM, STS, nc‐AFM and DFT calculations which suggests the following conclusions:

- The *ortho*‐position of halomethyl substituents in **DBN** and **TBN** ^[42]^ plays a significant role in reducing the activation barrier for debromination compared to the relevant *para*‐halogenated arenes. This generates on‐surface reactivity at room temperature which is so far rarely addressed in related on‐surface reactions after debromination;
- Though sharing a closely related molecular structures, the sequential reactions after debromination of **TBN** and **DBN** develop differently on Au(111) according to different intermediates. Their identification is crucial for understanding the competition of polymerization and dimerization;
- In solution, PPV and its derivatives are typically synthesized via precursor routes, that is, by polymerization of *p*‐quinodimethane intermediates and the subsequent polymer‐analogous β‐eliminations. In our case, such quinodimethane species are not observed. Instead, a vinylidene polymer without heteroatoms is observed which is then converted to conjugated poly(*o*‐naphthylene vinylene) through further dehydrogenation.

The present on‐surface syntheses together with the in situ visualization of intermediates and products helps to uncover a complex reactivity scheme which is still elusive in the analogous solution chemistry. We open here a promising prospect for developing dehalogenative homocoupling of halomethylarenes as well as other haloalkylarenes which offer the possibility to establish an extended conjugation by stereospecific dehydrogenation. We expect that the concept can serve as the guideline toward unprecedented conjugated polymers with adjustable electronic structures by varying aromatic cores and their connection. Future studies toward synthetic routes for organic electronics must address the question whether debromination of similar precursor molecules can occur on semiconducting or even insulating surfaces.

## Conflict of interest

The authors declare no conflict of interest.

1

## Supporting information

As a service to our authors and readers, this journal provides supporting information supplied by the authors. Such materials are peer reviewed and may be re‐organized for online delivery, but are not copy‐edited or typeset. Technical support issues arising from supporting information (other than missing files) should be addressed to the authors.

Supporting Information

## Data Availability

The data that support the findings of this study are available from the corresponding author upon reasonable request.
