# Supplementary material for: On‐Surface Debromination of 2,3‐Bis(dibromomethyl)‐ and 2,3‐Bis(bromomethyl)naphthalene: Dimerization or Polymerization?
Source: Angew Chem Int Ed Engl. 2022 Jun 8;61(30):e202204123. doi: 10.1002/anie.202204123 (PMC9401070; doi:10.1002/anie.202204123)
Supplement: Supplementary file 1 — Supporting Information [file ANIE-61-0-s001.pdf]

Angewandte Chemie
Eine Zeitschrift der Gesellschaft Deutscher Chemiker

Supporting Information

On-Surface Debromination of 2,3-Bis(dibromomethyl)- and 2,3-Bis(bromomethyl)naphthalene: Dimerization or Polymerization?

Y. Tang, B. Ejlli, K. Niu, X. Li, Z. Hao, C. Xu, H. Zhang, F. Rominger, J. Freudenberg, U. H. F. Bunz, K. Muellen, L. Chi**

Table of Contents

Table of Contents 2
Experimental Procedures 3
1.1 Reagents and Solvents for Synthesis 3
1.2 Column Chromatography 3
1.3 Nuclear Magnetic Resonance Spectroscopy 3
1.4 TGA/DSC 3
1.5 X-ray Single-Crystal Structure Analysis 3
1.6 On-Surface Characterization Parameters 3
Sample Preparation 3
STM/AFM and STS Measurements 3
1.7 Computational Parameters 3
Density Functional Theory (DFT) Calculations 3
AFM Images Simulations 4
2 Synthesis and Characterizations 4
2.1 Synthesis Route of **DBN** 4
2.2 Synthesis Route of **TBN** 5
2.3 Thermogravimetric Analysis and Differential Scanning Calorimetry of **DBN** and **TBN** 5
2.4 NMR Spectroscopy of **DBN** and **TBN** 6
2.5 Crystallographic Data 9
Results and Discussion 10
3.1 The Characterization of the Electronic Structure of Naphthocyclobutadiene (**3**) 10
3.2 Statistical Analysis of **TBN** Dimerization Products 10
3.3 Reaction of **DBN** on Au(111) in Low Coverage 11
3.4 Reaction of **DBN** on Pre-heated Au(111) 11
3.5 Polymerization and Dimerization Energy Barrier of **DBN** 12
References 12
Author Contributions 12

Experimental Procedures

1.1 Reagents and Solvents for Synthesis

All reagents and solvents were obtained from commercial suppliers (SigmaAldrich Laborchemikalien GmbH, TCI Deutschland GmbH) and used without further purification. Deuterated solvents for NMR analysis were purchased from SigmaAldrich Laborchemikalien GmbH.

1.2 Column Chromatography

Flash column chromatography was carried out using silica gel (grain size 0.04- 0.063 mm) produced by SigmaAldrich Laborchemikalien GmbH. As mobile phase the solvents named in the synthetic procedure were used. For thin layer chromatography Polygram Sil g/UV 254 plates from Macherey Nagel were used and examined under UV-light irradiation (254 nm and 365 nm).

1.3 Nuclear Magnetic Resonance Spectroscopy

All NMR spectra were recorded in deuterated solvents (CD_2Cl_2) at room temperature (if not stated otherwise) on a Bruker Avance III (300 MHz), Bruker Avance III (500 MHz) or Bruker Avance III (600 MHz). ^{13}C NMR spectra were measured proton decoupled if not stated otherwise. Chemical shifts δ are reported in part per million (ppm) and coupling constants J in Hz. All spectra were referenced to solvent signal.[1] For the multiplicities, the following abbreviations are used: s = singlet, d = doublet, t = triplet, m = multiplet. The spectra were processed and integrated using ACD/Spectrus processor.

1.4 TGA/DSC

Thermogravimetric analysis and differential scanning calorimetry- analysis were done using a Mettler Toledo TGA/DSC1 Star^e System. The ASCII files were exported and visualized by Origin Pro 2021.

1.5 X-ray Single-Crystal Structure Analysis

X-ray single-crystal structure analyses were measured on a Bruker Smart APEX-II Quazar Area detector. Diffraction intensities were corrected for Lorentz and polarization effects. An empirical absorption correction was applied using SADABS[2] based on the Laue symmetry of the reciprocal space. Hydrogen atoms were either isotropically refined or calculated. Structure was solved with SHELXT-2018/2 (Sheldrick 2015)[3] and refined against F2 with a Full-matrix least-squares algorithm using the SHELXL-2018/3 (Sheldrick, 2018)[4] software.

1.6 On-Surface Characterization Parameters

Sample Preparation

The Au(111)/Ag(111)/Cu(111) crystals that were purchased from MaTeck (Germany) and were cleaned through combined sputtering and annealing cycles. The precursors were deposited onto the single crystal surfaces by a commercial molecule beam evaporator purchased from Kentax (Germany). Reactions were triggered by annealing the sample at appropriate temperatures for 30 min. The samples were transferred into an STM/AFM scanner where the base pressure is better than 1.0×10^{-10} mbar.

STM/AFM and STS Measurements

All of the STM/AFM images were obtained at 4.6 K with a commercial LT-STM/AFM (Scienta Omicron, Germany). The tip was grounded, and the voltage bias was applied to the sample for STM imaging and STS characterization under the constant current mode. The STS spectra (dI/dV) were acquired using a lock-in amplifier (Vrms = 20 mV). The AFM images were obtained under constant-height mode by using a tuning fork force sensor with CO-functionalized tips. The resonance frequency of the force sensor is approximately 28 kHz. Vibration amplitudes of 50-69 pm were used for all the AFM images. The quality factors are 16 k-28 k.

1.7 Computational Parameters

Density Functional Theory (DFT) Calculations

All density functional theory (DFT) calculations were performed by the Vienna Ab-initio Simulation Package (VASP) together with Atomic Simulation Environment (ASE).[5] The electron-ion interactions were described by the projected augmented wave potentials and the Perdew-Becke-Ernzerhof functional (PBE) of generalized gradient approximation (GGA) was employed to treat exchange-correlation interactions.[6] The Van der Waals interactions were considered by the DFT-D3 method developed by Grimme.[7] The cutoff energy for the plane wave was set as 400 eV. The periodic image interactions were avoided by employing a 20 Å vacuum layer. The search for the transition states was conducted by a combination of Climb-Image Nudged Elastic Band (CI-NEB) method and Dimer

method.[8] First, 10 images were inserted in between the initial and final state by CI-NEB method. Second, the central image was further adopted as the input for the Dimer calculation in order to achieve high precision. The structure of local minima and the saddle points were optimized until the average atomic force was lower than 0.02 eV/Å. The coinage metal surfaces were modelled with periodic slabs with four atomic layers, in which atoms in bottom two layers were constrained. The Brillouin zone was modelled by gamma centre Monkhorst-pack scheme,[9] in which a 2 × 2 × 1 gird was used for all calculations.

AFM Images Simulations

The AFM simulation images are obtained on website: http://ppr.fzu.cz/. The Probe Particle Model is used in simulation of High-resolution atomic force microscopy (AFM), scanning probe microscopy (STM) and inelastic tunneling microscopy (IETS) images using classical forcefileds.[10] Files of three repeated units of polymer **12** and **4** were uploaded to obtain the AFM simulations. For both cases, the smallest tip heights were chosen for better resolution.

2 Synthesis and Characterizations

2.1 Synthesis Route of DBN

2,3-bis(bromomethyl)naphthalene (S2); **DBN**

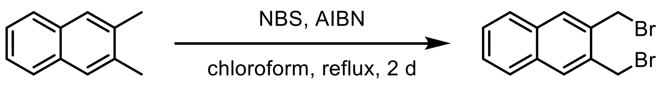

S1 **S2**

In a round-bottom flask, equipped with a reflux condenser, 2,3-dimethylnaphthalene (**S1**) (1.00 g, 6.40 mmol, 1.00 eq.) was dissolved in chloroform (50 mL). *N*-Bromosuccinimide (NBS) (2.34 g, 13.1 mmol, 2.05 eq) and azobisisobutyronitrile (AIBN) (50.0 mg, 320 µmol, 0.05 eq.) were added and the mixture was refluxed for 2 d. The reaction mixture was extracted with dichloromethane, washed with a saturated solution of sodium thiosulfate and dried over magnesium sulfate. The crude product was recrystallized from ethanol to obtain 2,3-bis(bromomethyl)naphthalene (**S2**) as colorless crystals (1.45 g, 4.62 mmol, 72%).

R_f = 0.58 (SiO_2; petroleum ether/ethyl acetate 9:1)

Mp: 145 °C

^{1}H NMR (CD_2Cl_2, 300 MHz, rt): δ = 7.89 (s, 2H), 7.82-7.85 (m, 2H), 7.51-7.55 (m, 2H), 4.90 (s, 4H) ppm.

^{13}C {^{1}H} NMR (CD_2Cl_2, 126 MHz, rt): δ = 134.4, 133.84 (C_q), 131.3, 128.2, 127.8, 31.71 (CH_2) ppm.

Data is in accordance with the literature.[16]

2.2 Synthesis Route of TBN

2,3-bis(dibromomethyl)naphthalene (S3); **TBN**

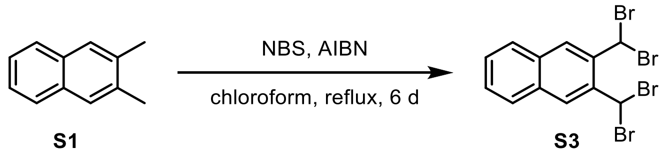

S1 **S3**

In a round-bottom flask, equipped with a reflux condenser, 2,3-dimethylnaphthalene (**S1**) (5.00 g, 32.0 mmol, 1.00 eq.) was dissolved in chloroform (250 mL). *N*-Bromosuccinimide (NBS) (28.5 g, 160 mmol, 5.00 eq.) and azobisisobutyronitrile (AIBN) (370 mg, 2.24 mmol, 0.07 eq.) were added and the mixture was refluxed for 6 d. The reaction mixture was extracted with dichloromethane, washed with a saturated solution of sodium thiosulfate and dried over magnesium sulfate. The crude product was purified via column chromatography (petroleum ether/ethyl acetate 2%) and recrystallized from ethanol to obtain 2,3-bis(dibromomethyl)naphthalene (**S3**) as colorless crystals (8.65 g, 18.3 mmol, 57%).

R_f = 0.51 (SiO_2; petroleum ether/ethyl acetate 9:1)

Mp: 158 °C

^{1}H NMR (CD_2Cl_2, 300 MHz, rt): δ = 8.22 (br s, 2H), 7.88-7.93 (m, 2H), 7.57-7.63 (m, 2H), 7.35 (br s, 2H) ppm.

^{13}C {^{1}H} NMR (CD_2Cl_2, 151 MHz, rt): δ = 132.8, 129.3, 128.9, 128.6, 37.9 ppm.

Data is in accordance with the literature.[17]

2.3 Thermogravimetric Analysis and Differential Scanning Calorimetry of **DBN** and **TBN**

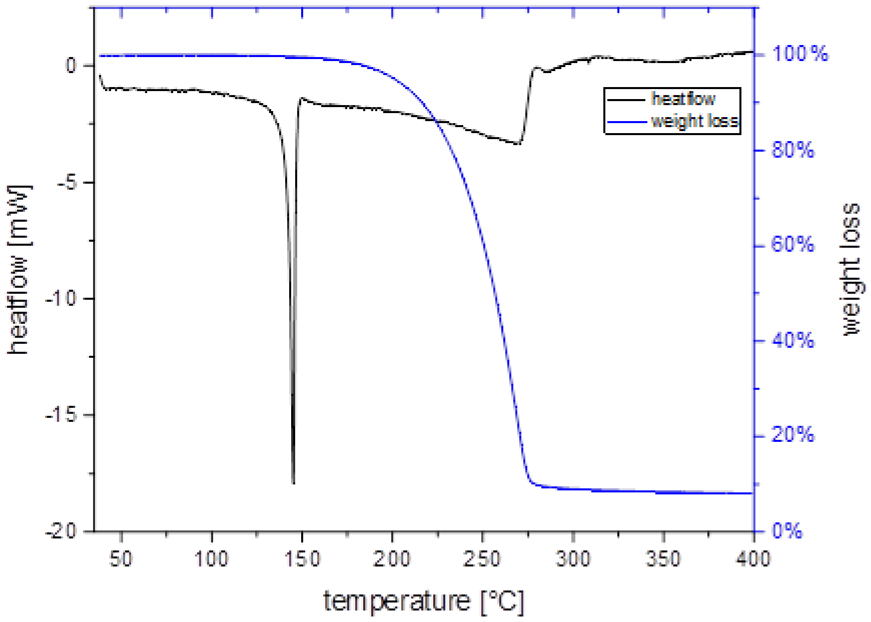

Figure S1: TGA/DSC measurement of 2,3-bis(bromomethyl)naphthalene **S2** (**DBN**).

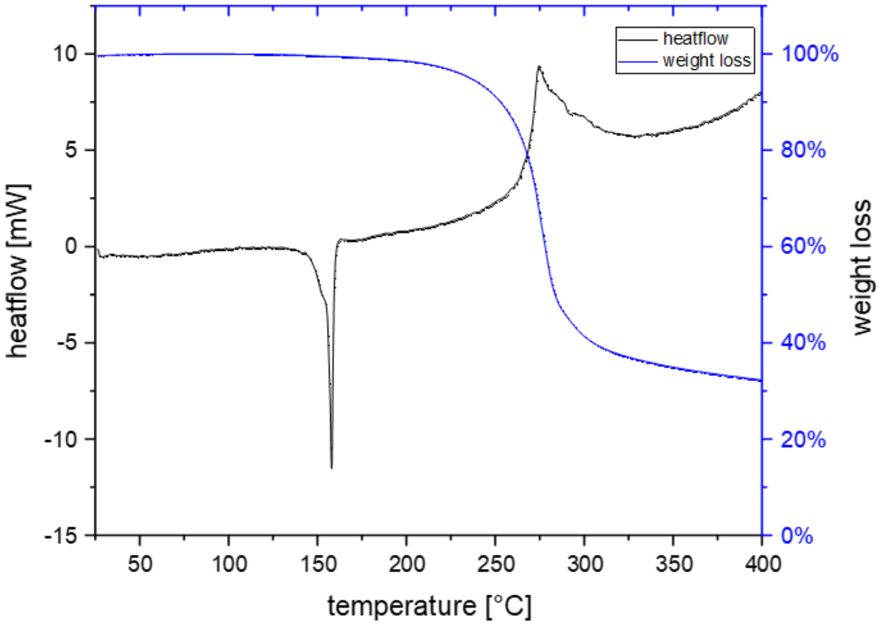

Figure S2: TGA/DSC measurement of 2,3-bis(dibromomethyl)naphthalene **S3** (**TBN**).

2.4 NMR Spectroscopy of DBN and TBN

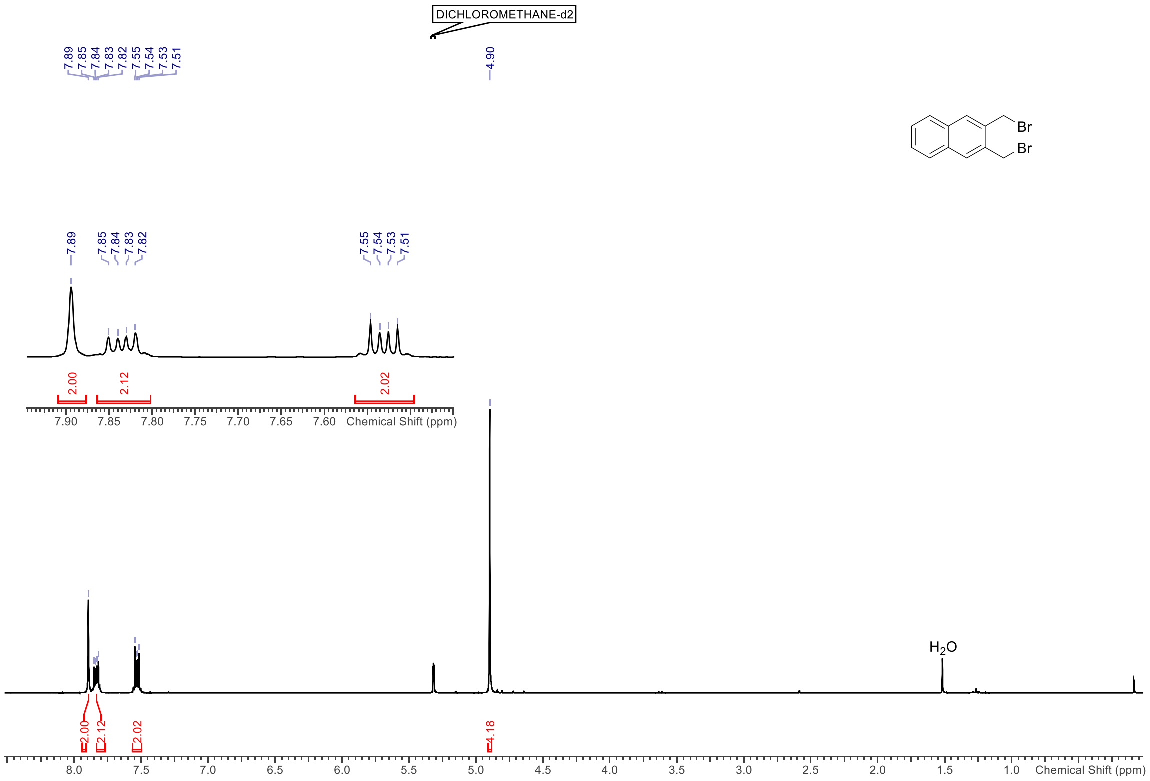

Figure S3: ^{1}H-NMR (300 MHz) of 2,3-bis(bromomethyl)naphthalene **S2** (**DBN**) in CD_2Cl_2.

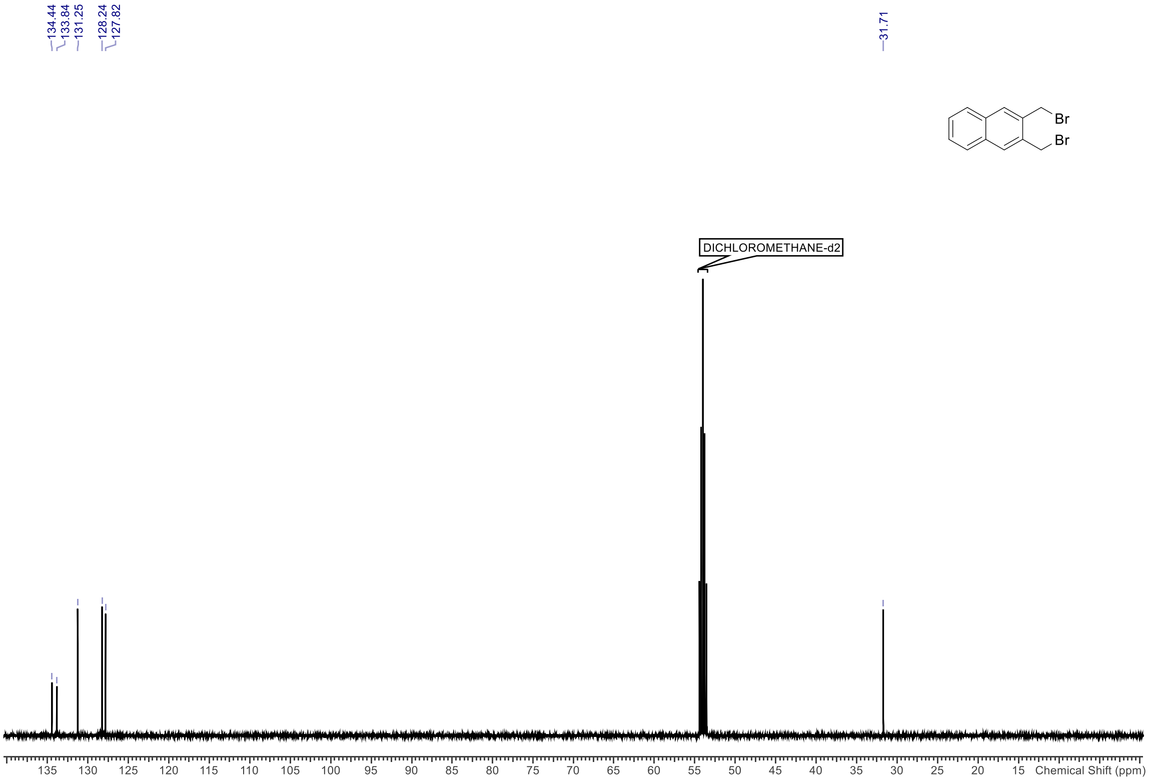

Figure S4: ^{13}C **{^{1}H}** -NMR (126 MHz) of 2,3-bis(bromomethyl)naphthalene **S2** (**DBN**) in CD_2Cl_2.

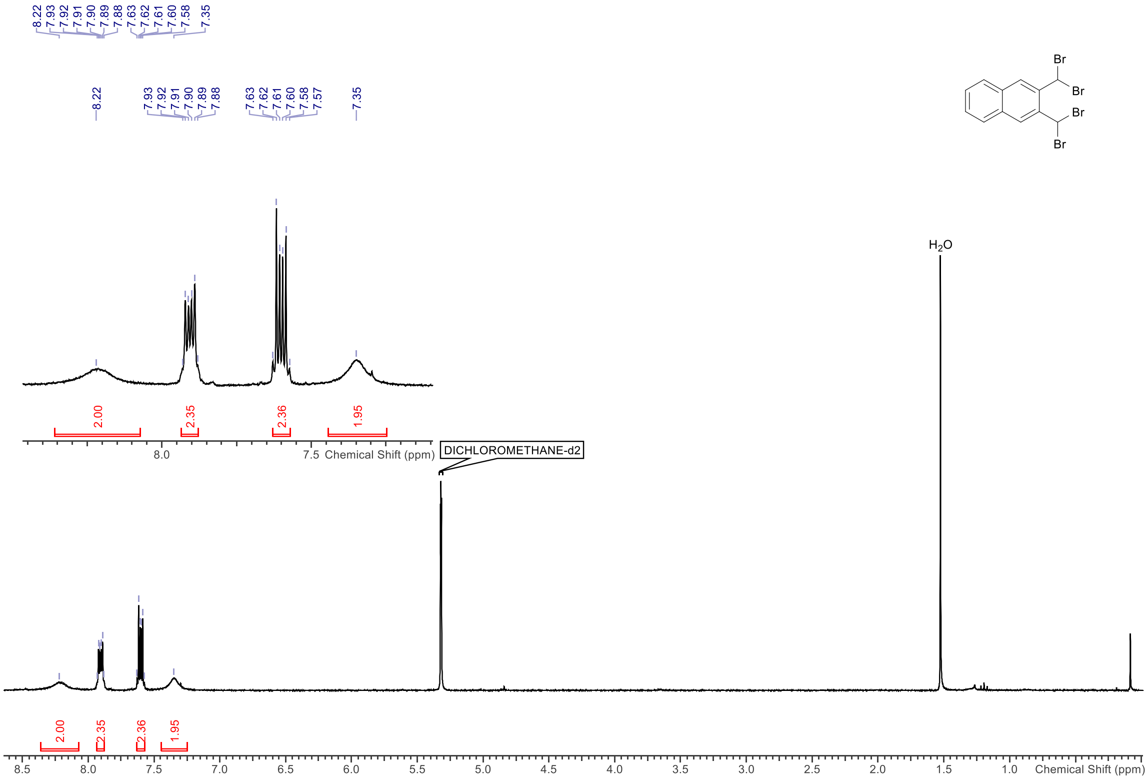

Figure S5: ^{1}H-NMR (300 MHz) of 2,3-bis(dibromomethyl)naphthalene **S3** (**TBN**) in CD_2Cl_2.

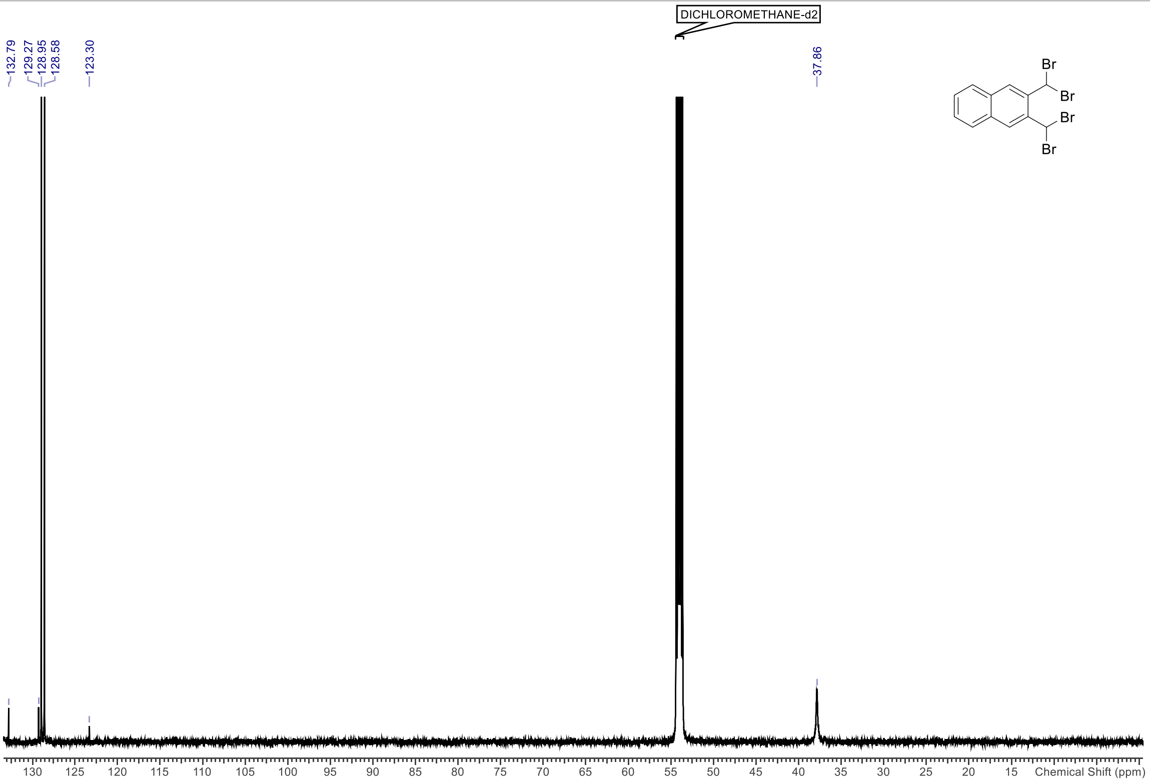

Figure S6: ^{13}C **{^{1}H}** -NMR (151 MHz) of 2,3-bis(dibromomethyl)naphthalene **S3** (**TBN**) in CD_2Cl_2.

2.5 Crystallographic Data

Table S1: Crystal structure, crystal data and structure refinement for **S3** (CCDC: 2099884)

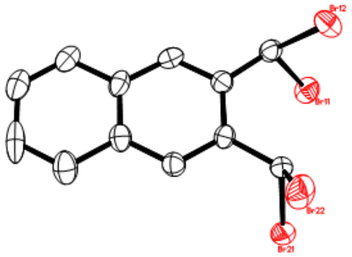

Empirical formula	$C_{12}H_8Br_4$	
Formula weight	471.82	
Temperature	200(2) K	
Wavelength	0.71073 Å	
Crystal system	monoclinic	
Space group	$P2_1/c$	
Z	4	
Unit cell dimensions	a = 10.5567(6) Å	α = 90 deg.
	b = 12.2984(6) Å	β = 96.5957(11) deg.
	c = 10.3205(5) Å	γ = 90 deg.
Volume	1331.05(12) $Å^3$	
Density (calculated)	2.35 g/cm^3	
Absorption coefficient	12.06 mm^{-1}	
Crystal shape	column	
Crystal size	0.153 x 0.028 x 0.015 mm^3	
Crystal colour	colourless	
Theta range for data collection	1.9 to 30.0 deg.	
Index ranges	-14≤h≤14, -17≤k≤16, -14εl≤14	
Reflections collected	17324	
Independent reflections	3893 (R(int) = 0.0349)	
Observed reflections	2902 (I > 2s(I))	
Absorption correction	Semi-empirical from equivalents	
Max. and min. transmission	0.86 and 0.61	
Refinement method	Full-matrix least-squares on F^2	
Data/restraints/parameters	3893 / 0 / 145	
Goodness-of-fit on F^2	1.03	
Final R indices (I>2sigma(I))	R1 = 0.039, wR2 = 0.086	
Largest diff. peak and hole	1.59 and -0.70 $eÅ^{-3}$	

Results and Discussion

3.1 The Characterization of the Electronic Structure of Naphthocyclobutadiene (3)

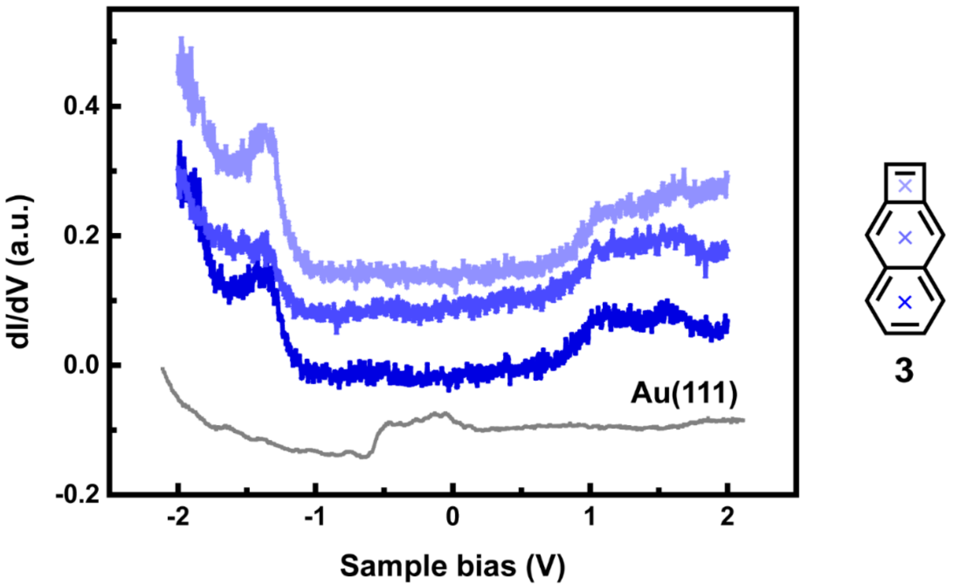

Figure S7: Full dI/dV spectra of naphthocyclobutadiene (**3**). Tip positions are marked by crosses in gradual change blue colours in corresponding position of **3**. STM onset set point (0.2 V, 10 pA) Spectra on Au(111) is first collected as reference spectra. The full dI/dV spectra of naphthocyclobutadiene (**3**) is collected to determine the HOMO and LUMO states. Tip positions do not make differences in spectra, indicating **3** is more of a comparatively conjugated structure electronically. Two prominent peaks are observed in each dI/dV curves. Peak certered at -1.35 V is considered as the HOMO state of **3**.

3.2 Statistical Analysis of TBN Dimerization Products

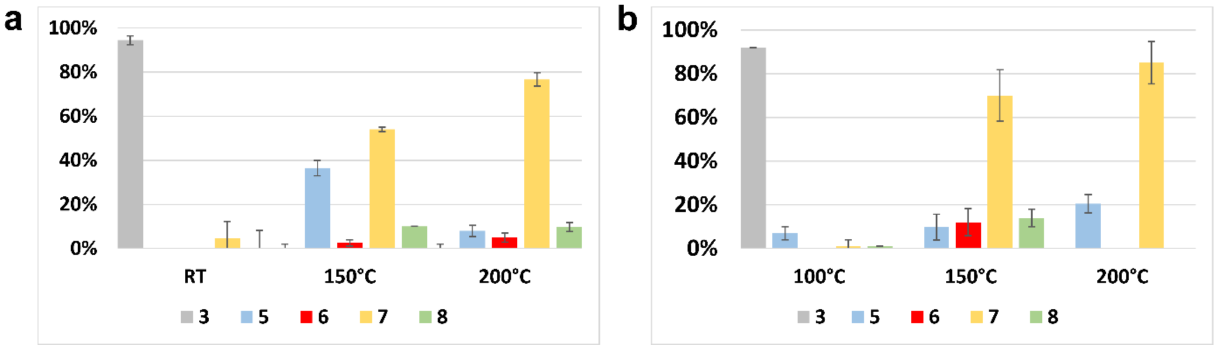

Figure S8. Statistical analysis of **TBN** dimerization products. (a) The samples are prepared on room temperature substrate and applied elevated annealing procedures. The proportion of each kind of dimer is counted and illustrated. (b) The samples are prepared on pre-heated substrate. The proportion of each kind of dimer is counted and illustrated.

3.3 Reaction of DBN on Au(111) in Low Coverage

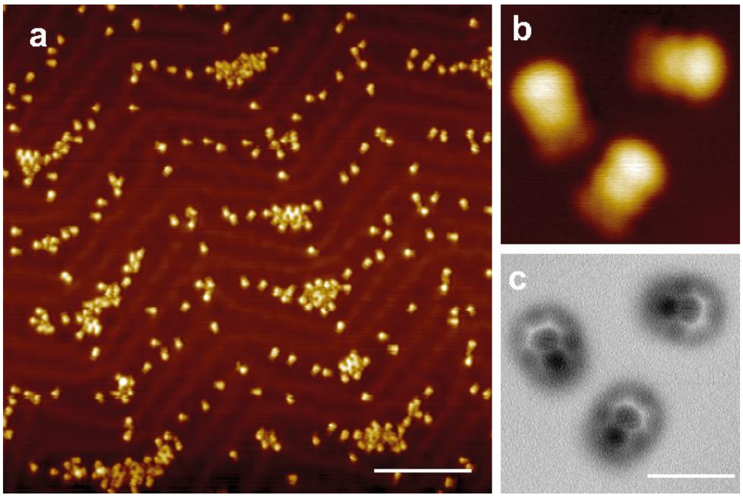

Figure S9. (a) STM image of DBN deposited on Au(111) in low coverage. (b, c) STM and AFM images of fully debrominated **DBN** (**10**). (I = 3 pA, V = -0.20 V, ΔZ= -190 pm) The white scale bars are 10 nm in (a) and 2nm in the rest of STM/nc-AFM images.

DBN is deposited on Au(111) surface hold at room temperature in low coverage. A debrominated intermediate state is observed. The AFM image reveals the nonplanar adsorption, demonstrating the debrominated radicals are bonded to the Au(111) surface.

3.4 Reaction of DBN on Pre-heated Au(111)

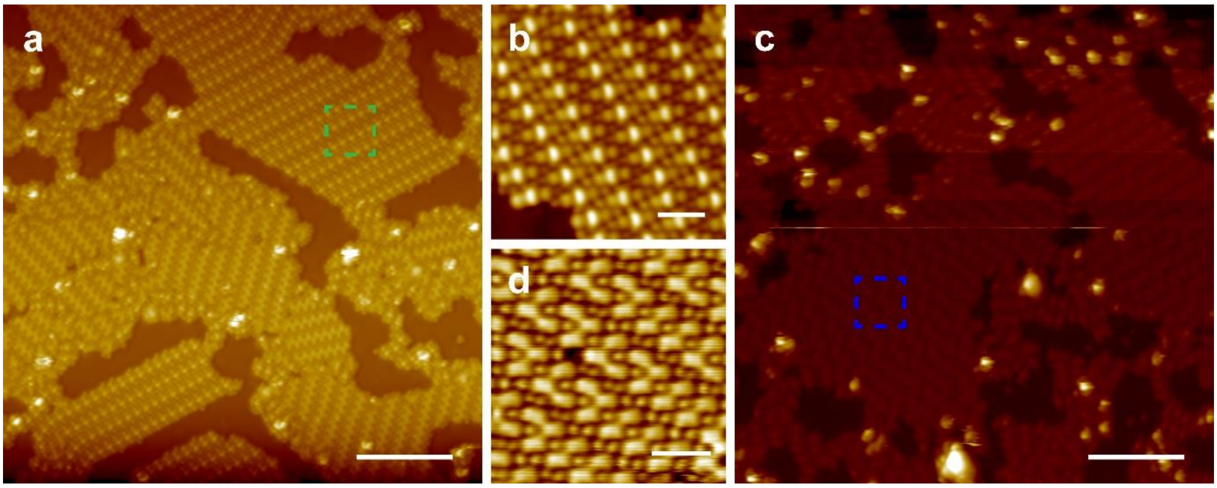

Figure S10. (a) STM image of **DBN** deposited on pre-heated Au(111) in 420 K. A green dashed square mark the area of major structure (dimer) (b) Zoom in STM image of dimers. (c) STM image of **DBN** deposited on pre-heated Au(111) in 470 K. A blue dashed square mark the area of major structure (oligomers) (d) Zoom in STM image of oligomeric species. The white scale bars are 10 nm in (a, c) and 2nm (b, d) of STM images.

3.5 Polymerization and Dimerization Energy Barrier of DBN

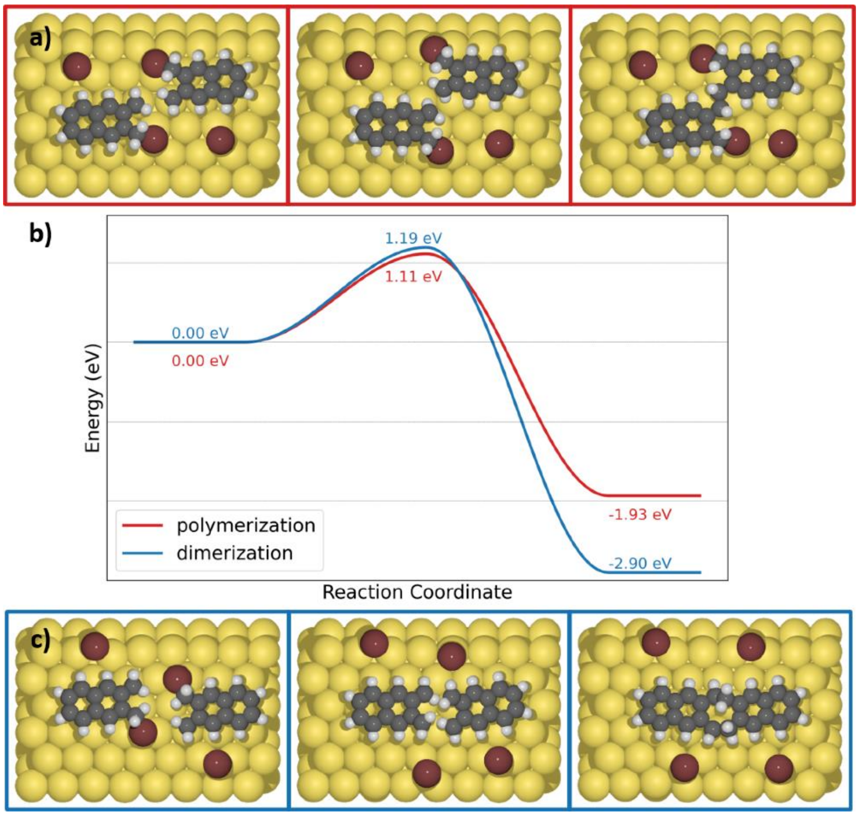

Figure S11. (a) Polymerization pathway of **DBN** on Au(111) at room temperature (labelled in red). (c) Dimerization pathway of **DBN** on Au(111) at room temperature (labelled in blue). (b) The corresponding energy profiles. The Au, Br, C and H atoms are represented by yellow, brown, grey, and white circles, respectively.

References

[1] G. R. Fulmer, A. J. M. Miller, N. H. Sherden, H. E. Gottlieb, A. Nudelman, B. M. Stoltz, J. E. Bercaw, K. I. Goldberg, *Organometallics* **2010**, *29*, 2176–2179.
[2] L. Krause, R. Herbst-Irmer, G. M. Sheldrick, D. Stalke, *J. Appl. Crystallogr.* **2015**, *48*, 3–10.
[3] G. M. Sheldrick, *Acta. Crystallogr. A Found. Adv.* **2015**, *71*, 3–8.
[4] G. M. Sheldrick, *Acta. Crystallogr. C Struct. Chem.* **2015**, *71*, 3–8.
[5] a) A. Hjorth Larsen, J. Jørgen Mortensen, J. Blomqvist, I. E. Castelli, R. Christensen, M. Dułak, J. Friis, M. N. Groves, B. Hammer, C. Hargus, E. D. Hermes, P. C. Jennings, P. Bjerre Jensen, J. Kermode, J. R. Kitchin, E. Leonhard Kolsbjerg, J. Kubal, K. Kaasbjerg, S. Lysgaard, J. Bergmann Maronsson, T. Maxson, T. Olsen, L. Pastewka, A. Peterson, C. Rostgaard, J. Schiøtz, O. Schütt, M. Strange, K. S. Thygesen, T. Vegge, L. Vilhelmsen, M. Walter, Z. Zeng, K. W. Jacobsen, *J. Phys. Condens. Matter.* **2017**, *29*, 273002; b) Kresse, Furthmüller, *Phys. Rev. B* **1996**, *54*, 11169–11186.
[6] a) P. E. Blöchl, *Phys. Rev. B* **1994**, *50*, 17953–17979; b) J. P. Perdew, K. Burke, M. Ernzerhof, *Phys. Rev. Lett.* **1996**, *77*, 3865–3868.
[7] S. Grimme, J. Antony, S. Ehrlich, H. Krieg, *J. Chem. Phys.* **2010**, *132*, 154104.
[8] a) G. Henkelman, H. Jónsson, *J. Chem. Phys.* **1999**, *111*, 7010–7022; b) G. Henkelman, H. Jónsson, *J. Chem. Phys.* **2000**, *113*, 9978–9985; c) G. Henkelman, B. P. Uberuaga, H. Jónsson, *J. Chem. Phys.* **2000**, *113*, 9901–9904.
[9] H. J. Monkhorst, J. D. Pack, *Phys. Rev. B* **1976**, *13*, 5188–5192.
[10] a) P. Hapala, G. Kichin, C. Wagner, F. S. Tautz, R. Temirov, P. Jelínek, *Phys. Rev. B* **2014**, *90*, 85421; b) P. Hapala, R. Temirov, F. S. Tautz, P. Jelínek, *Phys. Rev. Lett.* **2014**, *113*, 226101.

Author Contributions

Yanning Tang contributed to the STM/AFM data acquisition, data analysis and the writing of the original draft; Barbara Ejlli contributed to the synthesis and the analysis of precursors and the writing of the original draft; Kaifeng Niu contributed to the calculation and the writing of the original draft; Xuechao Li contributed to the data analysis and the writing of the original draft; Zhengming Hao contributed to the elementary STM experiments; Chaojie Xu contributed to the editorial work; Haiming Zhang contributed to the experimental administration; Frank Rominger contributed on the crystal structure elucidation; Jan Freudenberg contributed to editorial work and data analysis; Uwe H. F. Bunz contributed to editorial work and data analysis; Klaus Muellen contributed to initiate and administrate the project as well as to modify the manuscript; Lifeng Chi contributed to initiate and administrate the project, as well as to modify the manuscript

+ Yanning Tang, Barbara Ejlli, Kaifeng Niu and Xuechao Li contributed equally to this work.
